# Supplementary material for: Hybrid Sausages Using Pork and Cricket Flour: Texture and Oxidative Storage Stability
Source: Foods. 2023 Mar 16;12(6):1262. doi: 10.3390/foods12061262 (PMC10048543; doi:10.3390/foods12061262)
Supplement: Supplementary file 1 [file foods-12-01262-s001.zip › foods-2244678-supplementary.pdf]

Supplementary data

Table S1. Visual observation of gelation of cricket flour in water and NaCl solutions.

	**CF content (%, g/100 mL)**					
	1%	3%	5%	10%	20%	30%
Milli-Q	X A	X A	X A	O*	O	O
NaCl 1M	X A	X A	V	O	O	O
NaCl 2.1M	X A	X A	X A	O	O	O

CF: cricket flour

X: no gel formation

A: aggregation

V: viscous fluid

O: gel formation

O*: top layer, free and clear liquid/water was observed

Figure S1. Visual observation of sausage color

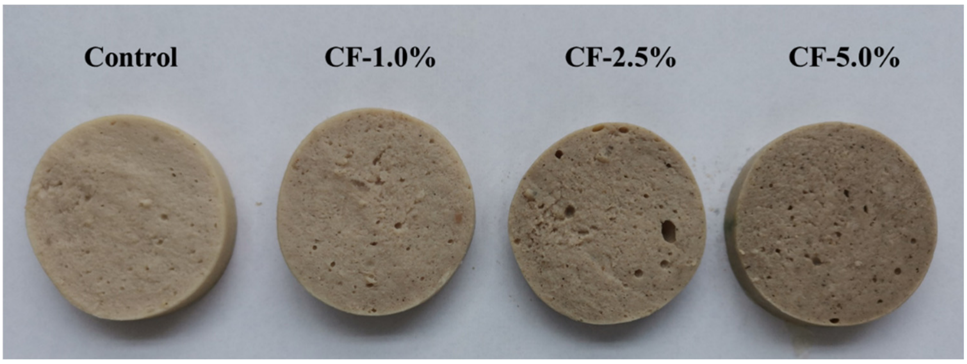

Table S2. Fatty acid composition of cricket flour, %

Common names		Mean	Std.Deviation
Lauric acid	C12:0	0.1 %	0.0 %
Myristic acid	C14:0	0.7 %	0.1 %
Myristoleic acid	C14:1	0.1 %	0.0 %
Palmitic acid	C16:0	34.9 %	0.0 %
Palmitoleic acid	C16:1	0.8 %	0.0 %
Margaric acid	C17:0	1.5 %	0.0 %
Stearic acid	C18:0	14.2 %	0.2 %
Oleic acid	C18:1	0.9 %	0.1 %
Linoleic acid	C18:2	45.4 %	0.2 %
Linolenic acid	C18:3	1.4 %	0.0 %
	ΣSFA	51.5 %	0.3 %
	ΣMUFA	1.8 %	0.1 %
	ΣPUFA	46.8 %	0.2 %

ΣSFA: Total saturated fatty acids

ΣMUFA: Total monounsaturated fatty acids

ΣPUFA: Total polyunsaturated fatty acids

Figure S2. Fatty acid composition of cricket flour, bar chart

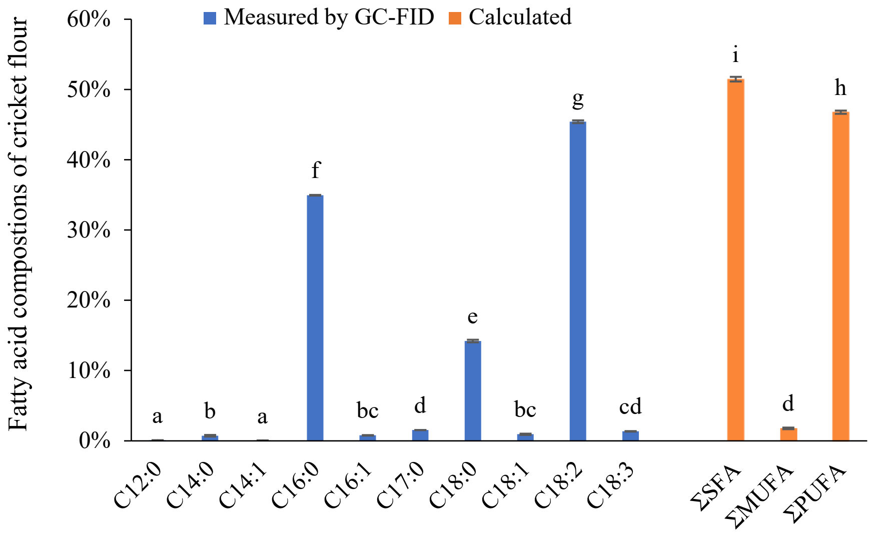

Significance differences ($p < 0.05$) are denoted by different lowercase letters a, b, c, d, e, f.
